# Decision-making efficiency with aided information: the impact of automation reliability and task difficulty

**DOI:** 10.1186/s41235-025-00659-w

**Published:** 2025-07-30

**Authors:** Hanshu Zhang, Ran Zhou, Cheng-You Cheng, Sheng-Hsu Huang, Ming-Hui Cheng, Cheng-Ta Yang

**Affiliations:** 1 Key Laboratory of Adolescent Cyberpsychology and Behavior (CCNU), Ministry of Education, 152 Luoyu Road, Hongshan District, Wuhan, 430079 Hubei China; 2 Key Laboratory of Human Development and Mental Health of Hubei Province, School of Psychology, Central China Normal University, Wuhan, 430079 China; 3 School of Psychology, South China Normal University, Guangzhou, 510631 China; 4 Department of Psychology, National Cheng Kung University, No.1, University Road, Tainan City, 701, Taiwan; 5 Department of Education and Humanities in Medicine, Taipei Medical University, No.250, Wu-hsing St., Taipei, 11031 Taiwan

**Keywords:** Aided decision-making, Automation reliability, Decision efficiency, Systems factorial technology

## Abstract

Although it is commonly believed that automation aids human decision-making, conflicting evidence raises questions about whether individuals would gain greater advantages from automation in difficult tasks. Our study examines the combined influence of task difficulty and automation reliability on aided decision-making. We assessed decision efficiency by employing the single-target self-terminating (STST) capacity coefficient in Systems Factorial Technology, estimating the ratio of performance with aided information to that without it. Participants were instructed to perform a shape categorization task, wherein they assessed whether the presented stimulus belonged to one category or another. In Experiment 1, three automation reliability conditions (high reliability, low reliability, and unaided) were tested in separate blocks. Our results indicated that, in general, participants exhibited unlimited capacity when provided with valid automated cues, implying that the decision efficiency was unaltered by automated assistance. Despite the failure to gain extra efficiency, the benefits of automated aids in decision-making for difficult tasks were evident. In Experiment 2, various types of automation reliability were randomly intermixed. In this scenario, the impact of automation reliability on participants’ performance diminished; however, the significance of information accuracy increased. Our study illustrates how the presentation of automation, its reliability, and task difficulty interactively influence participants’ processing of automated information for decision-making. Our study may improve processing efficiency in automated systems, hence facilitating superior interface design and automation execution.

## Significance statement

Decision makers engage with automation in intricate systems. Nevertheless, findings regarding the benefits of automated information in difficult tasks have been inconsistent. Our study explores whether these discrepancies arise from the ambiguous information on automation reliability. Using the single-target self-terminating (STST) capacity coefficient—a theory-driven tool for assessing decision efficiency—we evaluate the processing efficiency of unaided versus aided decision-making. The impact of task difficulty is evident when automation enables decision makers to gather reliable information effectively. However, this effect diminishes when the information regarding automation reliability is difficult to accumulate. Our findings indicate a potential synergy between task difficulty and automation reliability, which may enhance decision efficiency with automated assistance. This contributes to a deeper understanding of human-automation collaboration and improves interface designs in automated systems.

## Introduction

Humans are required to interact with automation in complex and complicated systems (Sheridan & Parasuraman, 2005). Comprehending the factors that affect the effectiveness of interaction, therefore, is essential for enhancing human-automation collaboration. Nevertheless, research remains ambiguous about the extent to which individuals gain benefits from automation, especially in the context of difficult tasks. Our current study examines the interaction between task complexity and automation reliability on decision efficiency, employing Systems Factorial Technology (hereafter SFT; Little et al., 2017; Townsend & Nozawa, 1995) for a detailed analysis of automated aids’ effect on human decision-making processes. We aim to elucidate how decision makers react to differing degrees of automation reliability across various task situations.

### Task difficulty and aided performance

While automation is often seen as beneficial for decision makers in both easy and difficult tasks (e.g., Chen et al., 2018), there is little agreement on its actual benefits in challenging scenarios. Some studies suggest that automation enhances performance in difficult tasks (Xu et al., 2007), while others observe a decline in problem-solving capabilities when automation is introduced (Bowers et al., 1998). Using workload capacity analysis within the framework of SFT, Kneeland et al. (2021) found no evidence that decision makers performed better with combined automated support under varying task difficulty conditions. This finding introduces a third perspective: Task difficulty may not influence the efficiency of aided decision-making.

Regardless of whether automation consistently yields performance benefits, previous research has often observed an increased reliance on automation in difficult tasks. This trend is partly attributed to the cognitive workload being offloaded by the presence of automated support. For instance, participants demonstrated higher accuracy performance when aided by highly reliable automation, especially under conditions of high cognitive load (Gamble et al., 2018). Similarly, reliance on automation increased under high time pressure (Hoff & Bashir, 2015). However, as previously discussed, increased reliance does not necessarily translate to improved performance. Rieger and Manzey (2022) found that while aided information helped mitigate some negative effects of time pressure, joint human-automation performance still remained inferior to automation-only performance.

The decision to rely on automation is further shaped by numerous other factors. Decision makers who are confident in their abilities may mistakenly believe that they do not need automated support for difficult tasks (Patton, 2023). This confidence trade-off can also reveal their belief in the task’s importance. Schwark et al. (2010) found that in visual search tasks, participants were more likely to use automated aids when tasks were explicitly labeled as difficult, regardless of the actual difficulty level. However, participants tended to rely more on their abilities when tasks labeled as difficult were associated with higher rewards, underscoring the influence of perceived importance on the utilization of automation.

### Trust toward the imperfect automation

Among the factors influencing the willingness to utilize automation, the most extensively discussed was automation reliability. Reliability is a critical sub-component of trust (Hoff & Bashir, 2015; Lee & Moray, 1992; Schaefer et al., 2016), and automated aids that demonstrate high reliability are more likely to be perceived as trustworthy. Decision makers who utilize highly reliable automation exhibited superior accuracy and response time performance compared to those using less reliable ones (Gamble et al., 2018). By contrast, poor automation can even worsen existing problems rather than alleviate them. Metzger and Parasuraman (2005) found that decision makers’ performance with aids was even worse than in the unaided condition. Despite these potential drawbacks, decision makers often choose to rely on flawed automation to preserve cognitive resources for other tasks. Indeed, when reliability falls below a critical threshold (in the range of 70–75%), automation may lead to increased errors and inefficiencies, perhaps yielding inferior performance compared to the absence of automation (Wickens & Dixon, 2007).

In decision-making contexts, decision makers often underestimate the actual reliability of imperfectly reliable automation (Chen et al., 2018; Wiegmann et al., 2001). This tendency may stem from a “perfect automation” schema, wherein decision makers expect flawless performance. As a result, automation errors can disproportionately influence decision makers’ estimates of aid reliability and reduce their willingness to rely on automation—despite evidence that aided diagnosis may outperform unaided diagnosis (Wiegmann et al., 2001). In a simulated warehouse management task, Barg-Walkow and Rogers (2016) examined perceptions of automation reliability. Participants were told that the automation was either 60%, 75%, or 90% reliable, although all groups experienced the same actual reliability of 75%. Participants in the 60% and 75% conditions underestimated the automation’s reliability, while those in the 90% condition provided more accurate estimations. Similarly, Pop et al. (2015) investigated perception of automation reliability in a simulated X-ray task, manipulating initial and subsequent reliability levels (100–80%, 60–80%, or 80–80%). The results indicated that participants adjusted their perceived reliability in response to changes in the actual performance of automation, with decision makers who had high expectations of automation’s reliability being more sensitive to changes. Interestingly, contrary to the assumption that a more accurate perception of automation reliability should lead to better performance and increased error detection, Merritt et al. (2015) found no evidence that decision makers’ adjustments in perceived reliability and trust in response to changes in the aid’s actual reliability over time significantly predicted task performance or the ability to identify automation failures.

In addition to the underestimation of automation’s reliability, self-confidence, which often varies across tasks, is a particularly influential factor in shaping trust formation (Lee & See, 2004). Decision makers frequently hold an inflated perception of their competence, especially when tasks appear easy, leading them to overestimate their capabilities (Dunning et al., 2003). This overconfidence can bias individual toward favoring their capabilities over automated support. For example, Kantowitz et al. (1997) found that when self-confidence exceeds trust in automation, decision makers were more likely to disregard automated advice—particularly in familiar contexts—and rely instead on their own decisions. Similarly, De Vries et al. (2003) also found that the difference between measures of trust and self-confidence was highly predictive of control allocation. In addition, their results also suggested a fundamental bias to trust one’s own abilities over those of the system. Notably, excessive reliance on automation also presents challenges. Highly reliable automation can lead to “complacency,” where decision makers neglect to monitor the information provided (Parasuraman & Wickens, 2017). The risks associated with complacency become more pronounced as automation reliability increases, as failures in highly reliable systems can result in more severe consequences (e.g., Rovira et al., 2007), underscoring the delicate balance required in managing trust and reliance on automation.

### The present study

The (mis)perception of task difficulty or the reliability of automation can lead to critical issues that maladaptively affect goal achievement during the decision-making process. Previous research has suggested that increasing task difficulty would amplify the influence of automation reliability (Wickens, 2000). Reliance on automation is more likely in situations where the automation is highly reliable and manual operation is effortful or cognitively demanding (Dzindolet et al., 2003; Wickens, 2002). Conversely, automation failures on easy tasks tend to have a more pronounced negative impact on trust and reliance, as such errors are more noticeable compared to difficulty tasks (Dzindolet et al., 2003; Madhavan et al., 2006).

Our research investigates the interplay between the task difficulty and automation reliability and their impact on processing efficiency in aided decision-making. Specifically, the automation is presented as an aided decision support system that provides recommended answers to decision makers (e.g., Rieger & Manzey, 2022). In Experiment 1, participants were instructed to perform a shape categorization task where the automation in high and low reliability provided aided information in the easy and difficult tasks. We hypothesize that the judgments with greater task difficulty will encourage decision makers to rely more on automation, and thereby, the automation reliability has a larger impact on decision-making efficiency. In contrast, for easy tasks, automation reliability may have minimal influence on its utilization.

To further examine the role of perceived automation reliability, Experiment 2 introduced three randomly presented automations with varying reliability levels, whereas the task difficulty was in a block design as in Experiment 1. We predict that when the automation reliability is difficult to assess, the impact of task difficulty on automation usage will become more pronounced. Following the methodology of Kneeland et al. (2021), our current research employs capacity analysis in SFT to compare decision-making performance with automated assistance to a null model in which decision makers performed tasks without aided information.

## Experiment 1

### Methods

#### Participants

Thirty-six university students (Ages [18, 28], *N*_female_ = 19) from National Cheng Kung University participated in the experiment. Participants signed the informed consent before the start of the experiment. The study was approved by the Human Research Ethics Committee of National Cheng Kung University (Protocol code: NCKU HREC-E109-257-2). Participants received NT$528 (approximately USD 16.5) as reimbursement for their total 3-h participation in the study.

#### Experimental apparatus

The experiment was conducted using a computer equipped with an Intel Core i7-8700 CPU running at 3.20 GHz. The experiment was programmed using MATLAB (MathWorks Inc.) and Psychtoolbox (http://psychtoolbox.org/), running on a 19-inch LED monitor with a screen resolution of 1920 × 1080 pixels and a refresh rate of 75 Hz.

#### Design, stimulus, and procedure

**Fig. 1:**
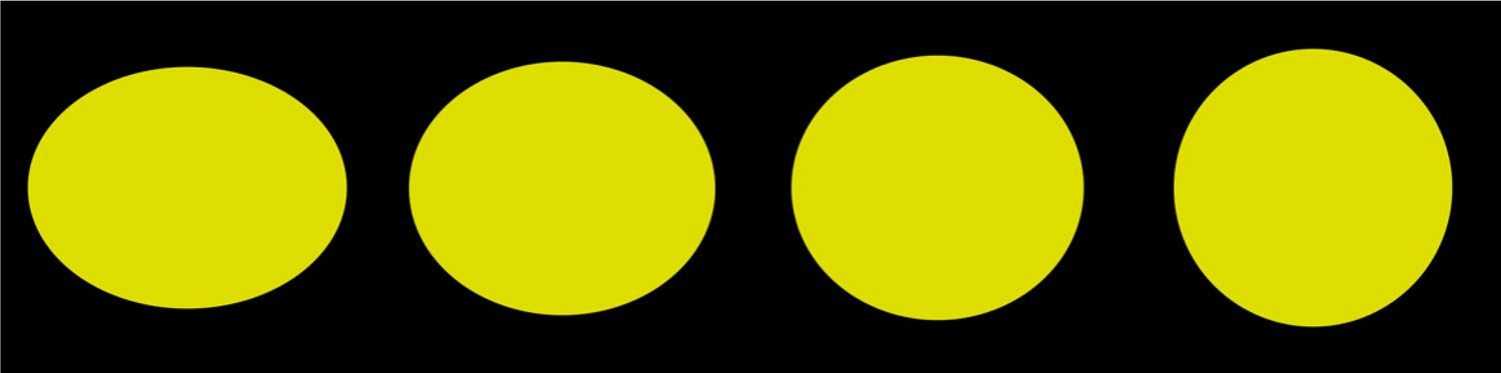
The stimuli in the study were in combinations of different categories and difficulty levels. From left to right: Category B (easy), Category B (difficult), Category A (difficult), Category A (easy)

Figure 1 depicts stimuli employed in the study that the stimulus transient from an ellipse-like shape to a circle (subtended visual angle from left to right: 0.53° × 0.72°, 0.51° × 0.75°, 0.48° × 0.79°, 0.46° × 0.81°), in which the left two stimuli were arbitrarily categorized as category A and the right two stimuli as category B. In addition, the middle of the two stimuli was considered more difficult to categorize.

Participants were instructed to decide as quickly as possible the category of the presented stimuli. In addition to the presented stimuli, participants were also provided with the automation suggestion (explained further below) when they were in the condition of the automated aid. In the *high-reliability condition*, the overall accuracy for the automation was 82%, and in the *low-reliability condition*, the overall accuracy for the automation was 50%, represented by different automation suggestion displays. That is, given a stimulus A, 82 out of 100 trials that the high-reliability automation provided valid suggestions, whereas 50 out of 100 trials that the low-reliability automation provided valid suggestions. A detailed explanation can be found in the Appendix. This resulted in a 2 (task difficulty: easy, difficult) × 3 (automation reliability: high, low, unaided) within-subject design.

**Fig. 2:**
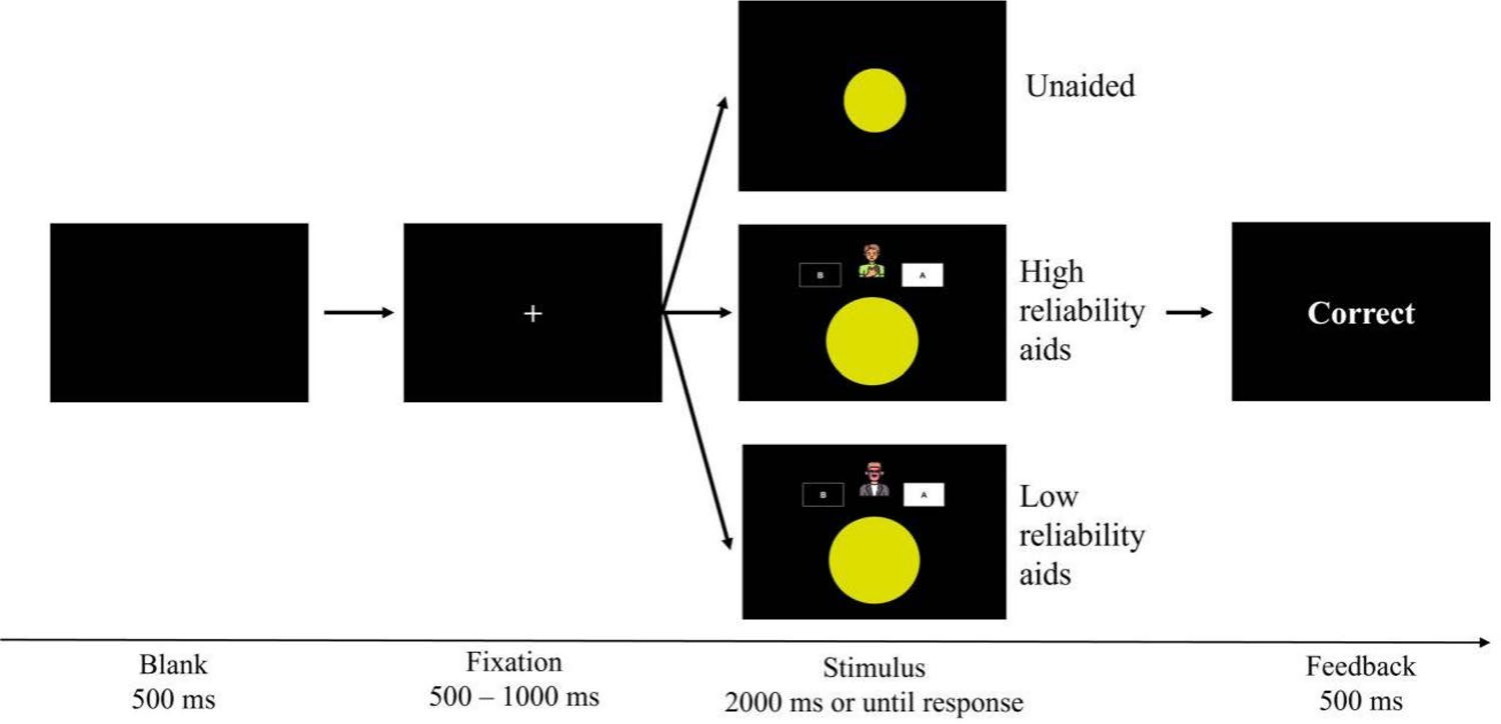
The procedure of a test trial. In the automated presented session, participants were also given the aids’ suggestions

The experiment is structured into three sessions, each focusing on a different automation reliability condition, with each session lasting approximately one hour. The test order was counterbalanced across participants. Figure 2 depicts a trial for the categorization task. Each trial began with a blank screen, followed by a fixation cross displayed at the center of the screen, ranging from 500 to 1000 ms. Then the test stimulus was presented for participants to make a judgment for 2000 ms or until a response. Participants were instructed to press “J” if the presented stimulus was (more closely resembled) a circle (category A) or “F” if the stimulus was (closely resembled) an ellipse (category B) as quickly and accurately as possible. During the response period in sessions featuring high or low-reliability automated aids, an automated aid (displayed as an “avatar”) appeared above the stimulus, with each type of reliability represented by a different avatar, offering classification suggestions to the participants. Participants received feedback after each trial.^1^ Each session included 400 trials, randomized across difficult and easy tasks. In total, each participant accomplished 1200 trials for the whole experiment.

#### Capacity coefficient

In the current research, we applied workload capacity within the framework of systems factorial technology (SFT; Townsend & Nozawa, 1995) to address the shortcomings of the traditional efficiency indicators that are merely implied by response times or accuracy. Workload capacity refers to the changes in decision efficiency as a function of workload, which provides a suite of response-based distributional measures for assessing changes in information processing efficiency when the aided information is provided. The single-target self-terminating (*C*_*STST*_, STST) stopping rule (Blaha et al., Under Review), a measurement for workload capacity, is calculated as a ratio:

$$C_{STST} \left( t \right) = \frac{{K_{unaided} \left( t \right)}}{{K_{aided} \left( t \right)}}$$

where *K* represents the cumulative reverse hazard function at time *t,* from both the aided and unaided trials. The cumulative reverse hazard function is represented by the natural logarithm of the cumulative distribution function (CDF), denoted as In[*F*(t)] = *K*(t), where *F*(t) stands for the CDF of response times. In this way, the ratio presents participants’ performance with the aided information compared to the unaided trials, during which participants have the option to either take into account the added information or disregard it during aided trials. The ratio was compared against a null model, which assumed the predicted performance based on an unlimited-capacity, parallel, and independent (UCIP) model (Townsend & Nozawa, 1995). The *C*_*STST*_ < 1 indicates participants of limited capacity, referring to that the decision efficiency declines when the automated aids are introduced. The super capacity,* C*_*STST*_ > 1, suggests that the presence of automated aids enhances decision efficiency. The *C*_*STST*_ = 1, on the other hand, suggests that there is no performance decrement or increment with the automated aids.

### Results

The goal of our current study was to examine the performance of participants when presented with and without the automated aids’ suggestions. However, a consistently low accuracy when the high-reliability aids produced incorrect responses (i.e., invalid cues) also indicated that participants did not actively engage in the task. In this way, four participants were excluded from further analysis because their accuracy was below 0.5 when high-reliability aids presented invalid cues for the difficult task. Additionally, trials with responses faster than 100 ms or slower than 1500 ms (approximately 0.88% of total trials) were excluded for further analysis. The behavior analysis in response times and mean accuracy was conducted in ANOVA using JASP (JASP Team, 2024). We use Greenhouse–Geisser for sphericity correction and Bonferroni correction for the post hoc tests. The SFT package (Houpt et al., 2013) in R was used for STST analysis.

#### RT

**Fig. 3:**
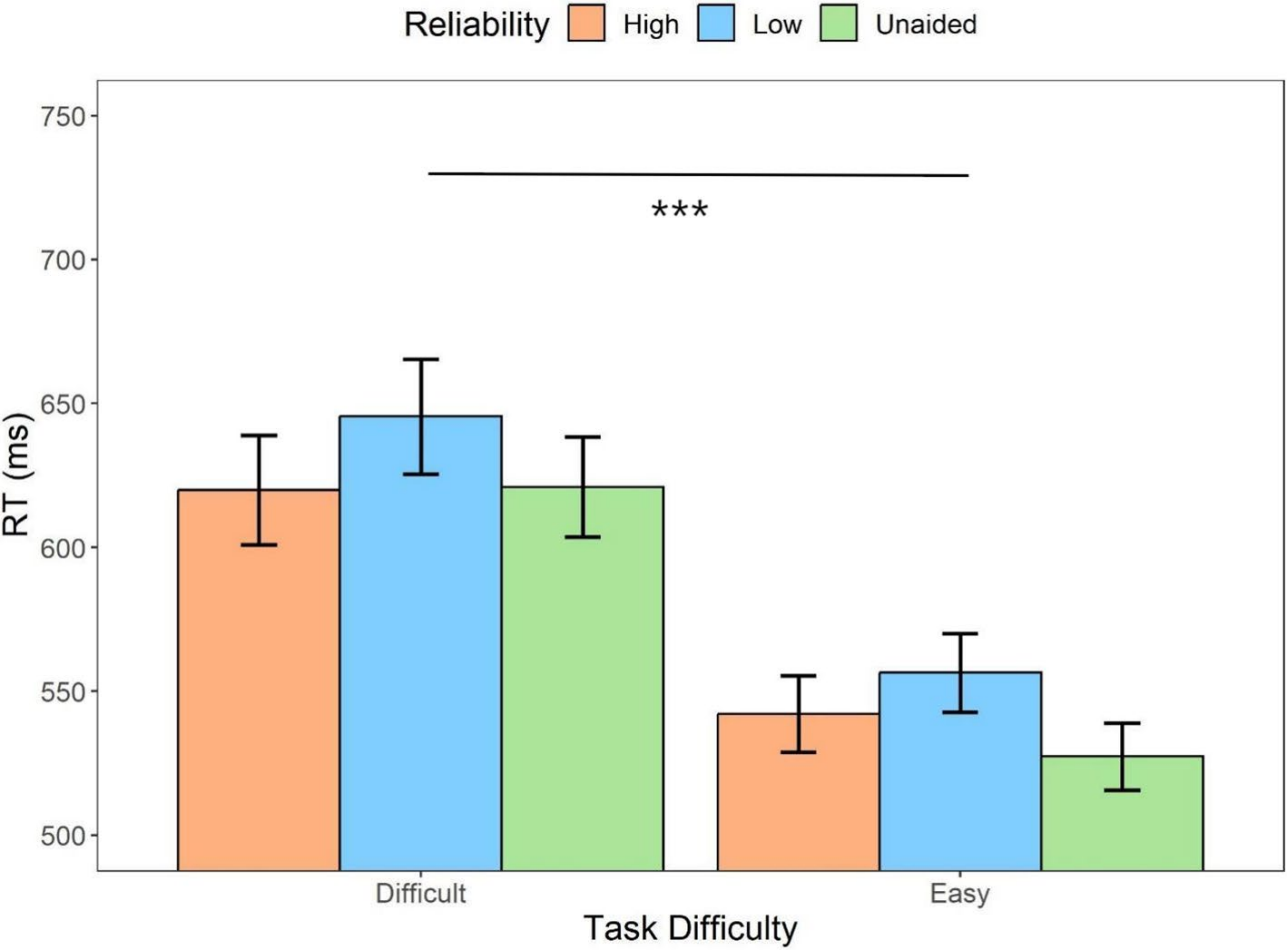
Mean response times across conditions of task difficulty (difficult, easy) and automation reliability (high, low, unaided) in Experiment 1. Error bars represent ± one standard-error

We first examined the influence of automation conditions (unaided, high reliability, low reliability) and task difficulty (difficult, easy) on the response times of correct responses. The repeated measures ANOVA (Fig. 3) indicated that there was a main effect of the automation condition (*F* (2,62) = 4.19, *p* = .02, *η*_*p*_^2^ = *.*12), indicating that the unaided condition led to significantly faster responses compared to the low-reliability-aided condition (*t* = 2.79, *p* = .02, Cohen’s *d* = 0.29). There was no statistical significance between the responses in the high-reliability-aided condition and low-reliability-aided condition (*t* = 2.07, *p* = .13, *d* = 0.22), as well as the unaided condition (*t* = 0.72, *p* = 1, *d* = 0.08). In addition, there was a main effect of the task difficulty (*F* (1,31) = 116.86, *p* < .001, *η*_*p*_^2^ = *.*79), participants responded faster to the easy task compared to the difficult task (*t* = 10.81, *p* < .001, *d* = 0.95). The results also indicated that there was an interaction between the two factors (*F* (2,62) = 3.75, *p* = .03, *η*_*p*_^2^ = .11): The reaction time (RT) discrepancy between the high-reliability automated condition and the unaided condition in difficult tasks (*M*_*diff*_ = -1.00, 95%CI [ -31.51, 29.52])) was notably smaller compared to the difference observed in easy tasks (*M*_*diff*_ = 14.84, 95%CI [-15.67, 45.36]). This suggests that decision makers responded even more quickly on their own in easier tasks.

**Fig. 4:**
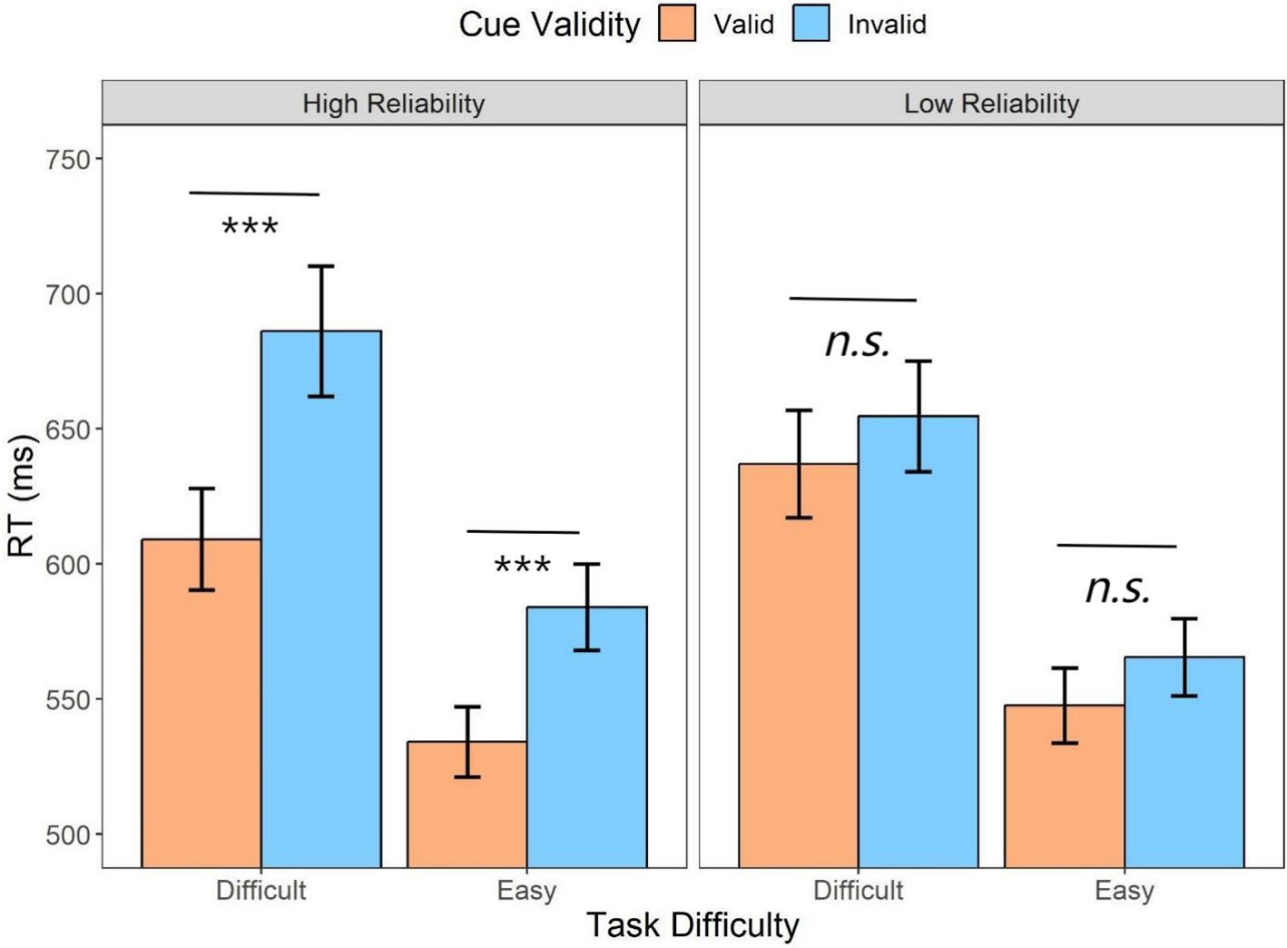
Mean response times across conditions of cue validity (valid, invalid) and task difficulty (difficult, easy) for different automation reliability (high, low) types in Experiment 1. Error bars represent ± one standard-error

Considering that automation reliability could lead to distinct attentional allocations, an additional categorization for the automated session was implemented based on whether the cue was valid to examine its impact on the participants’ response times. The repeated measures ANOVA (Fig. 4) on automation reliability (high, low), task difficulty (difficult, easy), and cue validity (valid, invalid) suggested that there were main effects of task difficulty (*F* (1,31) = 100.53, *p* < .001, *η*_*p*_^2^ = *.*76) and cue validity (*F* (1,31) = 37.24, *p* < .001, *η*_*p*_^2^ = *.*55): The difficult task resulted in slower responses (*t* = 10.03, *p* < .001, *d* = 0.88) compared to the easy task, and the slower responses were also exacerbated by invalid cues compared to valid cues (*t* = 6.10, *p* < .001, *d* = 0.40).

The results indicated that there was also a three-way interaction (*F* (1,31) = 6.34, *p* = .017, *η*_*p*_^2^ = *.*17). To further investigate the three-way interaction, the two-way repeated measures ANOVA on task difficulty and cue validity was conducted for the high- and low-reliability conditions, respectively. When the automation reliability was high, there was an interaction between the cue validity and task difficulty (*F* (1,31) = 7.36, *p* = .01, *η*_*p*_^2^ = *.*19), resulting from a larger RT difference between the valid cues and invalid cues when the task was difficult (*M*_*diff*_ = 77.02, 95%CI [42.56, 111.48]) compared to when the task was easy (*M*_*diff*_ = 49.84, 95%CI [15.38, 84.30]). However, such an interaction effect was not significant when the automation reliability was low (*F* (1,31) = 0.001, *p* = .97, *η*_*p*_^2^ < *.*001).

#### Accuracy

**Fig. 5:**
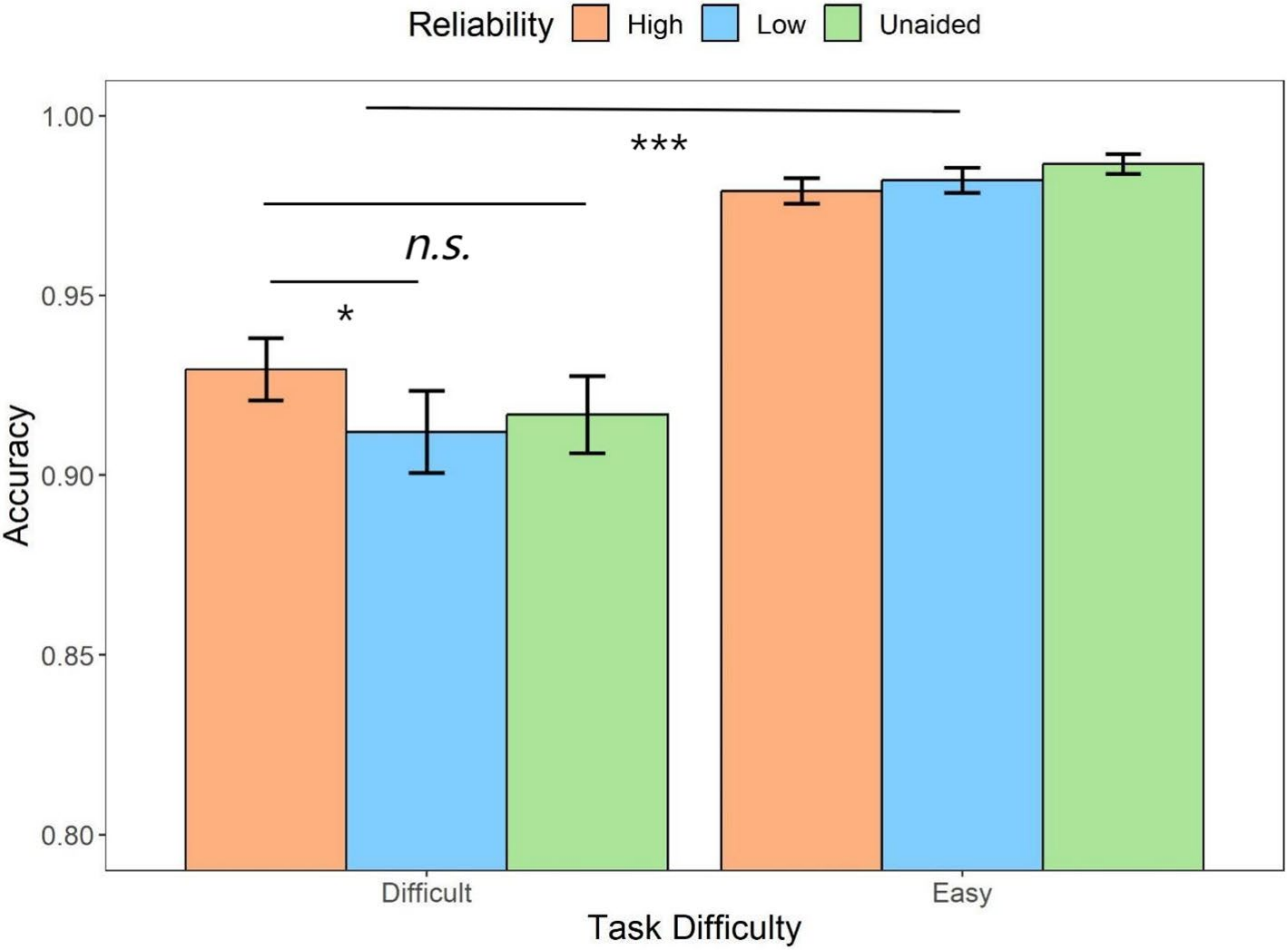
Mean accuracy across conditions of task difficulty (difficult, easy) and automation reliability (high, low, unaided) in Experiment 1. Error bars represent ± one standard-error

Similar to RT analyses, the repeated measures ANOVA on the automation reliability and task difficulty were conducted on the response accuracy (Fig. 5). There was a main effect of task difficulty (*F* (1,31) = 62.42, *p* < *.*001, *η*_*p*_^*2*^ = 0.67). Participants performed better in the easy task compared to the difficult task (*t* = 7.90, *p* < .001, *d* = 1.45). There was also an interaction between the two factors (*F* (1.57,48.58) = 4.84, *p* = *.*011, *η*_*p*_^*2*^ = 0.14), likely resulting in the higher accuracy lead by the high-reliability automation compared to the low-reliability automation (*M*_*diff*_ = 0.02, 95%CI [5.30 × 10^–4^, 0.03]) and unaided condition (*M*_*diff*_ = 0.01, 95%CI [-0.004, 0.03]) when the task was difficult whereas it led to relatively lower accuracy in the easy condition (high reliability vs. low reliability: *M*_*diff*_ = - 0.003, 95%CI [ − 0.02, 0.14]; high reliability vs. unaided: *M*_*diff*_ = -0.007, 95%CI [ -0.02, 0.009]).

**Fig. 6:**
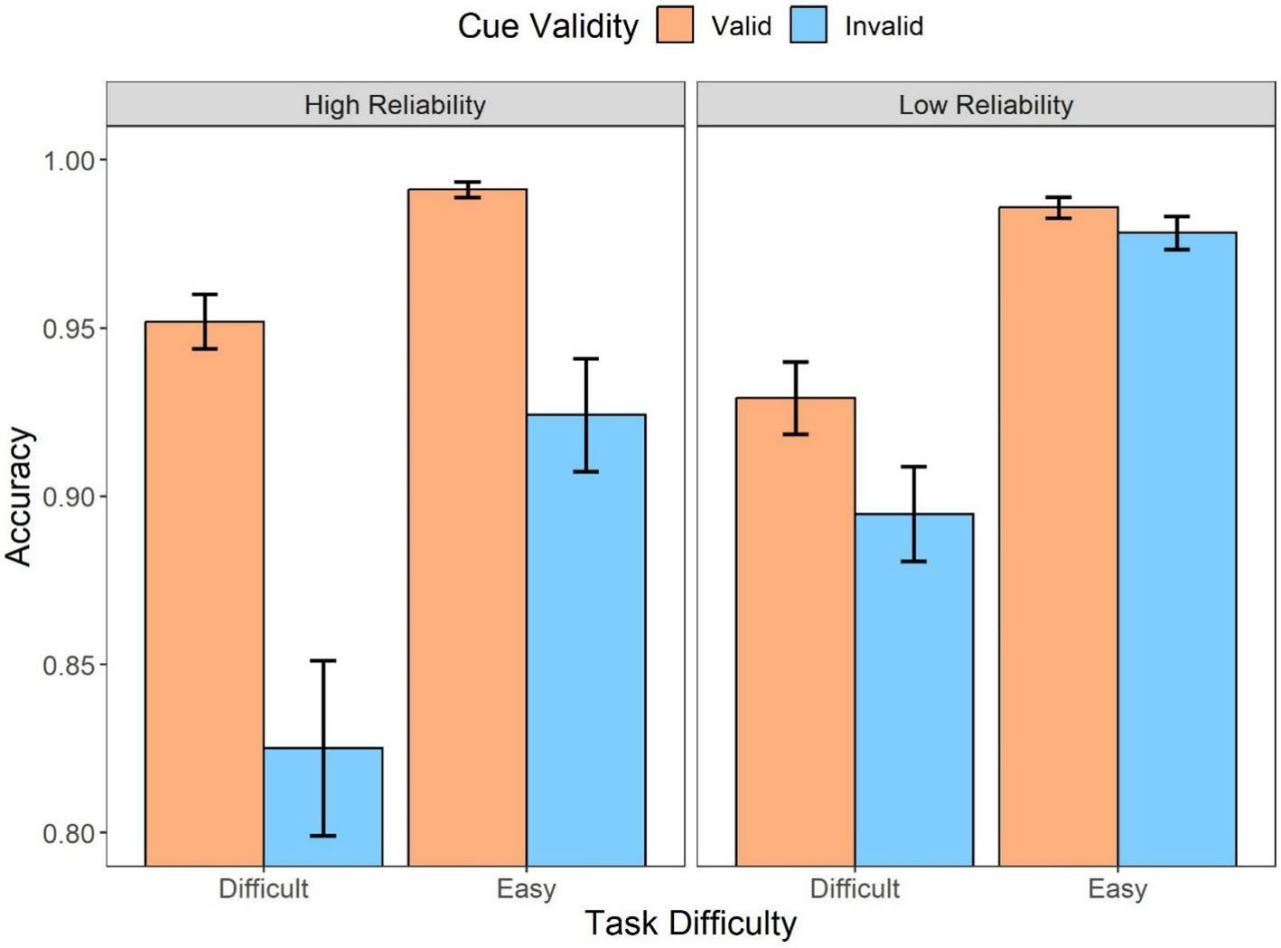
Mean accuracy across conditions of cue validity (valid, invalid) and task difficulty (difficult, easy) for different automation reliability (high, low) types in Experiment 1. Error bars represent ± one standard-error

The further analysis with cue validity included (Fig. 6) suggested that there were statistically significant main effects of the automation reliability (*F* (1,31) = 9.60, *p* = .004, *η*_*p*_^2^ = 0.24), task difficulty (*F* (1,31) = 44.39, *p* < .001, *η*_*p*_^2^ = 0.59), and cue validity (*F* (1,31) = 27.13, *p* < .001, *η*_*p*_^2^ = 0.47). The post hoc analysis indicated that low reliable automation (*t* = 3.10, *p* = .004, *d* = 0.32), easy task (*t* = 6.66, *p* < .001, *d* = 0.94), and valid cues (*t* = 5.21, *p* < .001, *d* = 0.79) were more likely to result in higher accuracy. Furthermore, there was an interaction between the automation reliability and cue validity (*F* (1,31) = 16.26, *p* < .001, *η*_*p*_^2^ = 0.34). When the automation was highly reliable, the valid cues produced significantly higher accuracy compared to invalid cues (*t* = 6.58, *p* < .001, *d* = 1.31) where such effect was not observed in the low-reliability condition (*t* = 1.43, *p* = .95, *d* = 1.43). In addition, the cue validity also interacted with the task difficulty (*F* (1,31) = 15.21, *p* < .001, *η*_*p*_^2^ = 0.33), suggesting that the accuracy difference between the valid and invalid cues (i.e., a larger validity effect) was observed only in the difficult task (*M*_*diff*_ = 0.08, 95%CI [0.05, 0.12], *t* = 6.39, *p* < .001) rather than in the easy task (*M*_*diff*_ = 0.04, 95%CI [0.002, 0.07], *t* = 2.96, *p* = .03).

#### Capacity coefficient

**Fig. 7:**
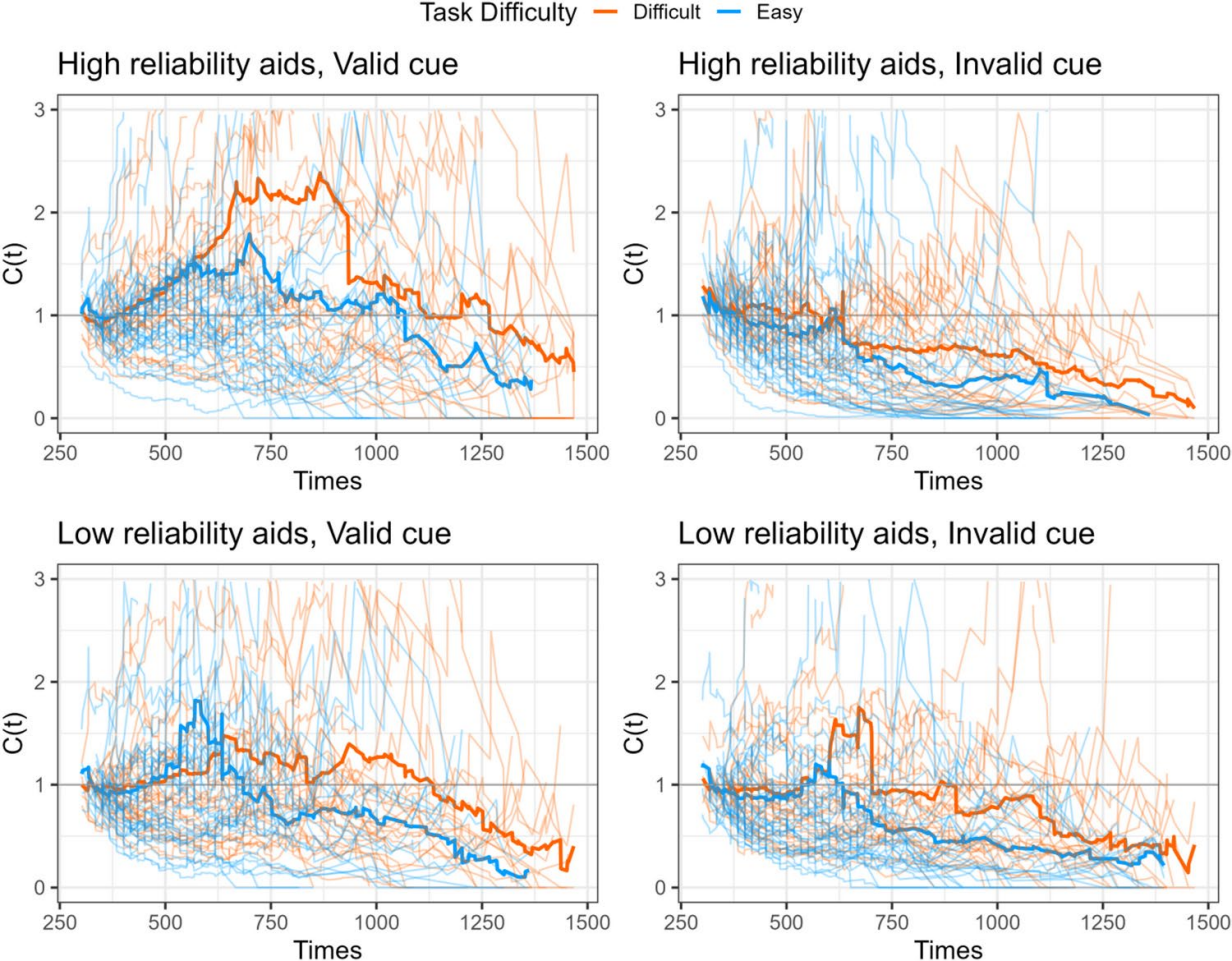
The temporal changes of capacity functions in Experiment 1. The thick line represents the average mean group level and the thin lines represent each individual’s capacity function

The STST capacity was calculated to indicate whether the aided information influenced processing efficiency compared to the predicted baseline based on the participant’s performance in the unaided decisions. Figure 7 displays the dynamic change of the capacity coefficient across response times. The visual inspection suggests that participants performed at limited to unlimited capacity. Notably, the figure also indicated that at the visual inspection, participants processed super capacity during the 500 ms and 750 ms period (Fig. 7, the left top panel), suggesting the facilitated processing aided by the automation.

**Fig. 8:**
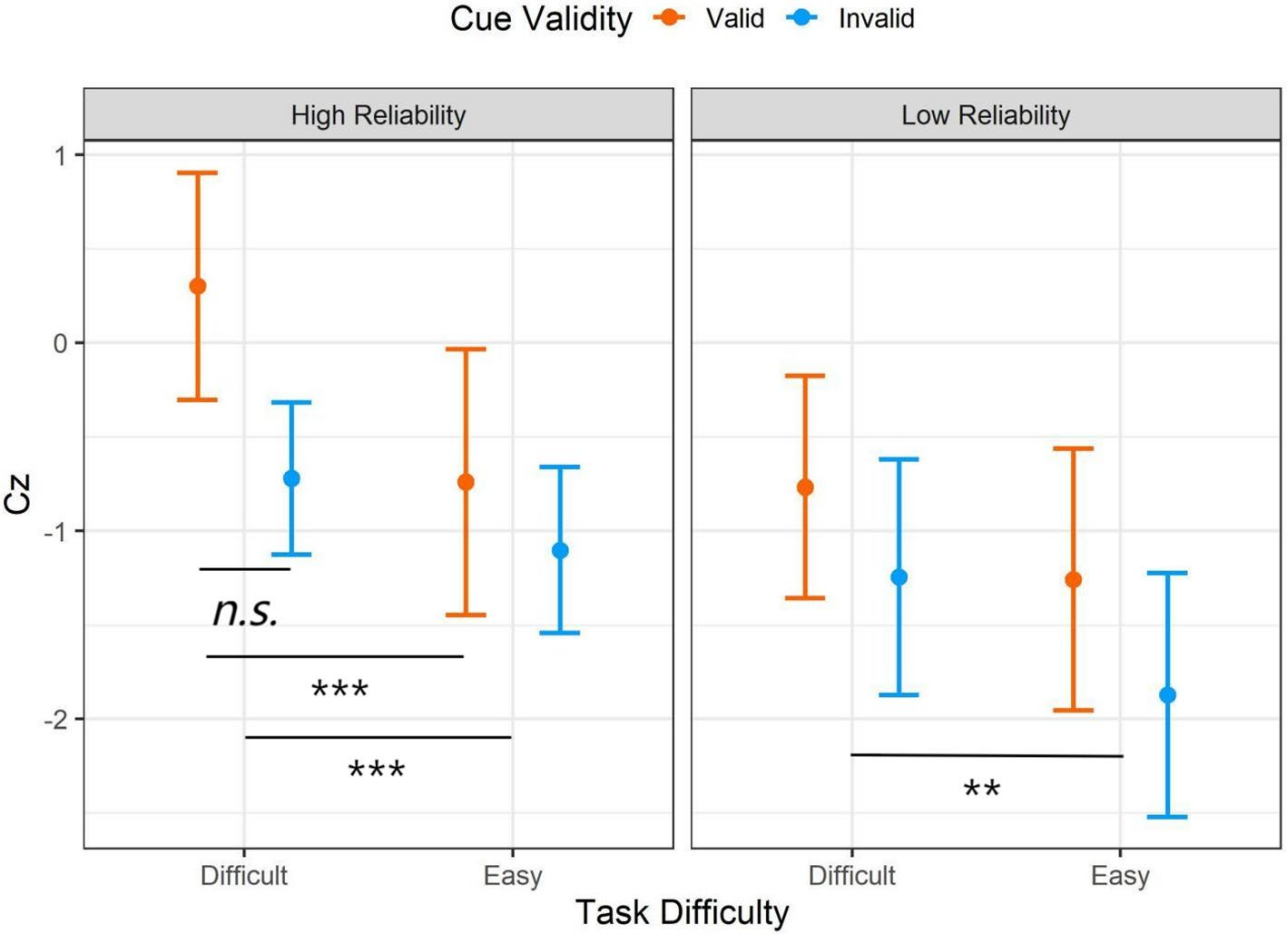
Mean Cz values for STST capacity coefficients for processing additional automated aids in Experiment 1. Cz at 0 implies unlimited capacity and below 0 implies limited capacity. The error bars represent ± one standard-error

To statistically quantify the observed capacity performance, the raw capacity scores are transformed to the statistic *Cz* to provide a summarized measure of processing efficiency across the entire response times as depicted in Fig. 8 (Houpt & Townsend, 2012). The summarized *Cz* scores are tested against 0, in which the *Cz* < 0 signals the limited capacity and *Cz* > 0 signals the super capacity. The one-sample *t-*test suggested the performance was unlimited capacity at the group level (i.e., not statistically significantly different from *Cz* = 0) except that the limited capacity performance when the automation presented the invalid cues in the easy task (high reliability: *M* = -1.10, *t*(31) = 2.52, *p* = .017, *d* = 0.45; low reliability: *M* =-1.87, *t* = 2.88, *p* = .007, *d* = 0.51).

The repeated measures ANOVA indicated that there was a main effect of the cue validity (*F* (1,31) = 8.05, *p* = .008, *η*_*p*_^2^ = 0.21), the valid cue resulted in higher processing efficiency compared to the invalid cues (*t* = 2.84, *p* = .008, *d* = 0.18). In addition, there was a main effect of the task difficulty (*F* (1,31) = 14.01, *p* < .001, *η*_*p*_^2^ = 0.31) where the aided decisions in a difficult task were more efficient than in an easy task (*t* = 3.74, *p* < .001, *d* = 0.20). There was also a three-way interaction (*F* (1,31) = 4.34, *p* = .045, *η*_*p*_^2^ = 0.12). The separate ANOVA analyses were conducted on the high- and low-reliability aids to further elucidate the three-way interaction. There was an interaction between the task difficulty and cue validity when participants performed in the high-reliability condition: There was a larger disparity in processing efficiency between the valid and invalid cues when the task was difficult (*M*_*diff*_ = 1.00, 95%CI [ -0.10, 2.11]) compared in the easy task (*M*_*diff*_ = 0.36, 95%CI [-0.75, 1.46]). The interaction between the task difficulty and cue validity, nevertheless, failed to display when the automation reliability was low.

### Discussion

In Experiment 1, participants were asked to perform a shape categorization task with aids (high reliability and low reliability) and without aids. The behavioral analysis indicated that the easy task resulted in faster response times and higher accuracy, validating our experimental design for difficulty manipulation. Additionally, consistent with previous research (Kneeland et al., 2021; Yamani & McCarley, 2018), our behavioral results also suggested that automated aids in the low-reliability condition resulted in slower responses compared to unaided condition, suggesting that the automated aids could potentially result in additional costs. One notable finding was the interaction between automation reliability and cue validity on accuracy. As we hypothesized, in situations where automation had high reliability, valid cues resulted in significantly higher accuracy compared to invalid cues, a distinction not observed in the low-reliability condition. This suggested that low reliability produced higher accuracy performance when the cue was invalid, demonstrating the complacency effect for the high-reliability automation, where overreliance led to a “neglect” of the “raw” stimuli information (Parasuraman & Wickens, 2017).

Furthermore, we performed the STST capacity analysis to examine the impact of automated aids on decision efficiency. The results demonstrated that participants operated at unlimited capacity across most combinations of reliability and task difficulty conditions, except for a noted limitation in capacity when automated aids supplied invalid cues during easy tasks. Contrary to other studies suggesting that participants may rely on aided information to save time (Chen et al., 2018), the observed unlimited-capacity performance demonstrated that automation neither improved nor diminished decision-making efficiency. Consistent with our hypothesis that automation reliability has a larger impact on difficult tasks, our results also demonstrated improved decision efficiency with aided information during difficult tasks compared to easy ones. To assess the extent to which task difficulty interacts with automation reliability, participants in Experiment 2 engaged in the categorization task with varied automated aids randomly presented. In this circumstance, we anticipated that the impact of task difficulty would be further intensified.

## Experiment 2

### Methods

#### Participants

A group of 36 university students (Ages: [19, 28], *N*_female_ = 18) from the same participant pool were recruited for Experiment 2. Similar to Experiment 1, participants signed the consent form before the experiment and they received NT$528 (approximately USD 16.5) as reimbursement for their participation.

#### Experimental procedure

In Experiment 2, participants were instructed to perform the same categorization task as used in Experiment 1. The key difference was that in Experiment 1, participants encountered only one type of automation reliability per session, while in Experiment 2, they were presented with three different types of automation reliability, which were displayed in a random order within each session.

### Results

The analysis conducted in Experiment 2 mirrored that of Experiment 1. Additionally, trials with response times below 100 ms or above 1500 ms were omitted from further analysis, accounting for about 1.7% of the total trials. This exclusion criterion was consistent with the approach taken in Experiment 1.

#### RT

**Fig. 9:**
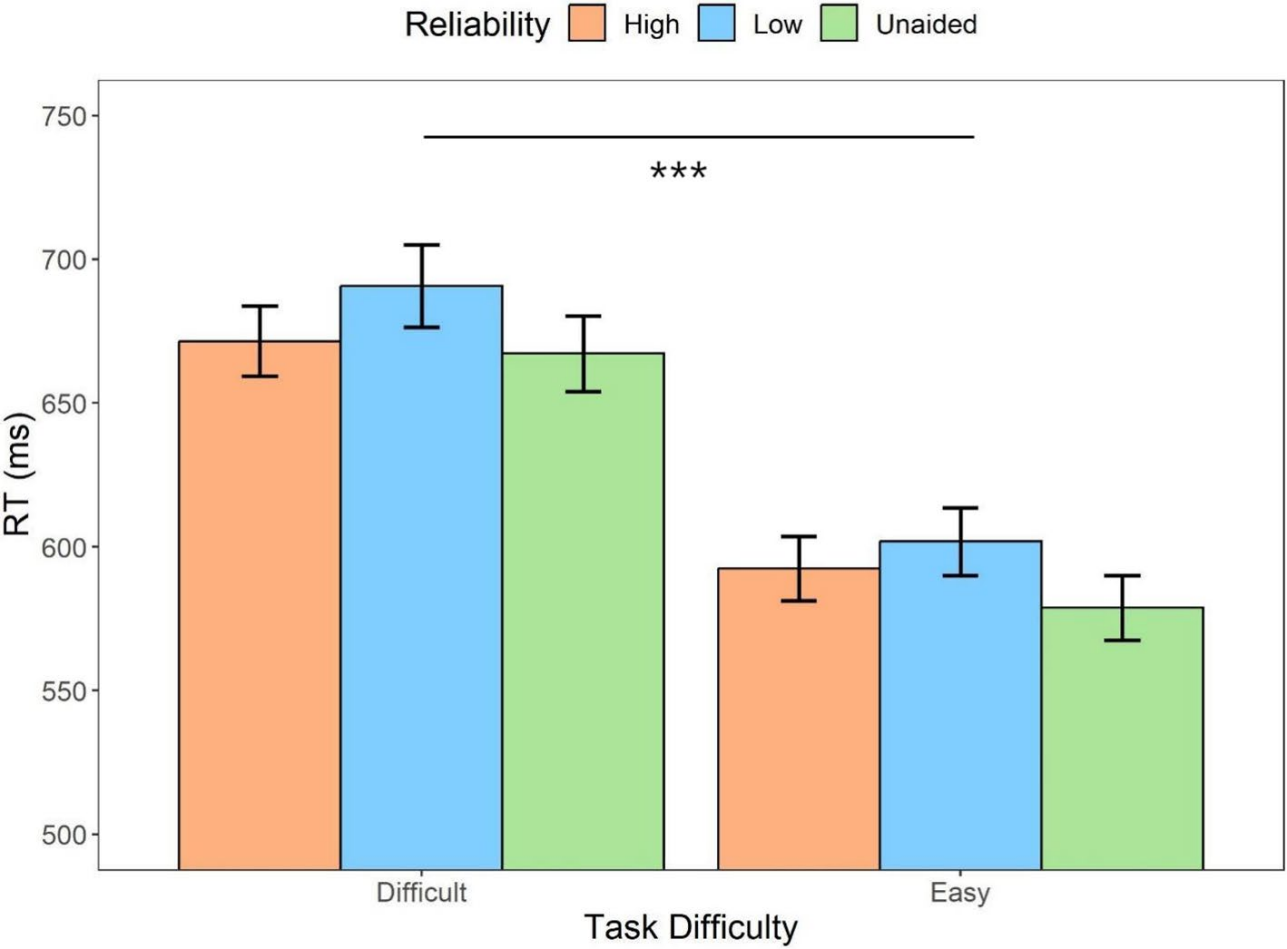
Mean response times across conditions of task difficulty (difficult, easy) and automation reliability (high, low, unaided) in Experiment 2. Error bars represent ± one standard-error

Similar to Experiment 1, we first explored the influence of task difficulty and automation reliability on response times for corrected responses. The repeated measures ANOVA (Fig. 9) suggested that there was a main effect of the automation reliability (*F* (1.59, 55.88) = 21.83, *p* < .001, *η*_*p*_^2^ = 0*.*38). The high-reliability automation resulted in faster responses than the low-reliability automation (*t* = 4.03, *p* < *.*001, *d* = 0.19). Unaided trials also resulted in a faster response than the high- (*t* = 2.53, *p* = .04, *d* = 0.12) and low-reliability-aided trials (*t* = 6.55, *p* < .001, *d* = 0.31), respectively. There was also a main effect of the task difficulty (*F* (1,35) = 280.26, *p* < .001, *η*_*p*_^2^ = *.*89); the difficult task resulted in slower responses compared to the easy task (*t* = 16.74, *p* < .001, *d* = 1.15). The results failed to indicate the interaction between the two factors (*F* (2,70) = 1.68, *p* = .19, *η*_*p*_^2^ = 0.05).

**Fig. 10:**
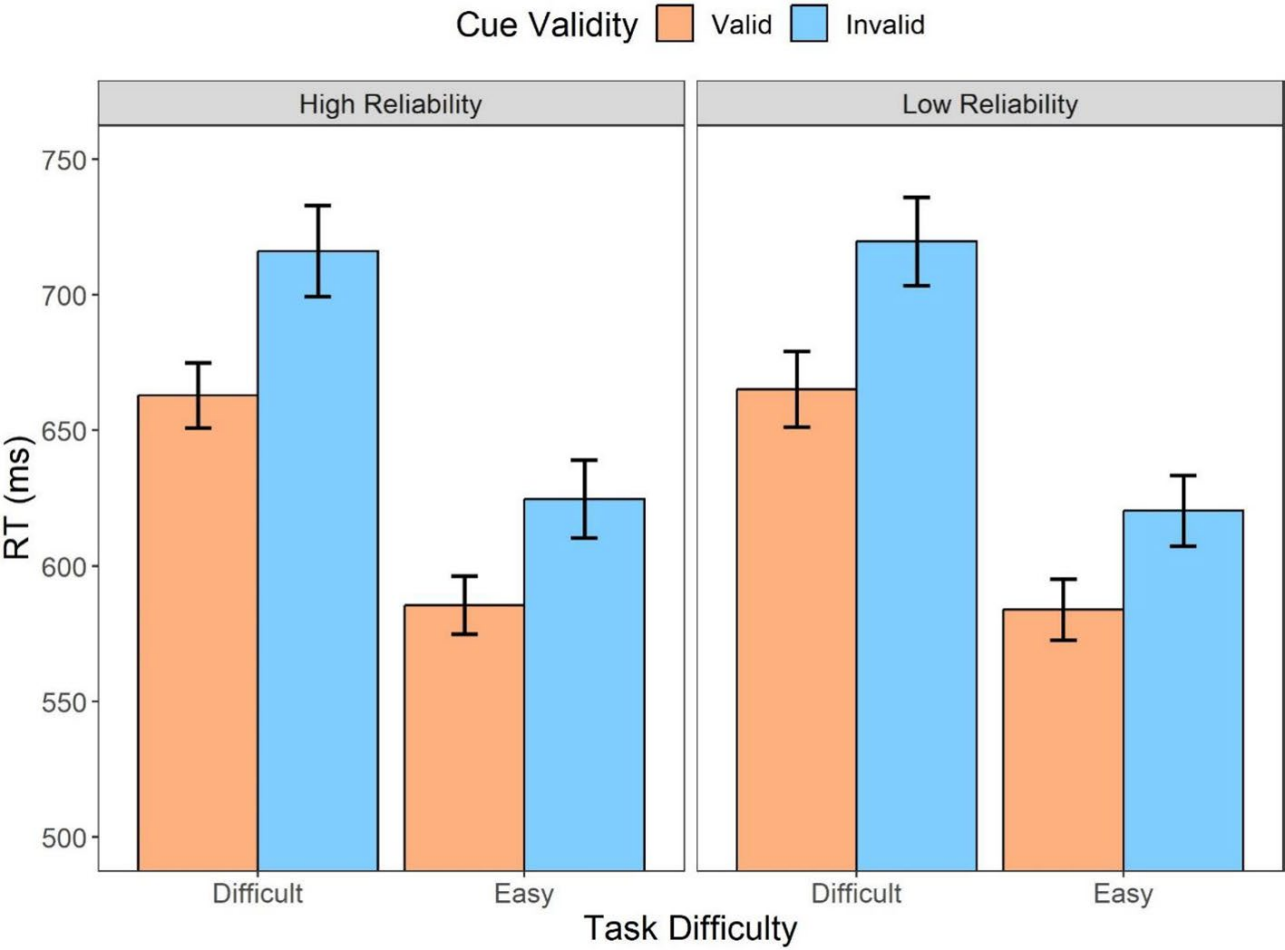
Mean response times across conditions of cue validity (valid, invalid) and task difficulty (difficult, easy) for different automation reliability (high, low) types in Experiment 2. Error bars represent ± one standard-error

Further analysis was conducted to investigate the influence of the cue validity (Fig. 10). The results indicated that there was a main effect of task difficulty (*F* (1,35) = 244.73, *p* < .001, *η*_*p*_^2^ = 0.88); the difficult task resulted in significantly slower responses compared to the easy task (*t* = 15.64, *p* < .001, *d* = 1.06). Furthermore, there was a main effect of the cue validity; the invalid cues resulted in significantly slower responses compared to the valid cues (*t* = 6.90, *d* = 0.56, *p* < .001). The analysis also indicated an interaction between the cue validity and task difficulty (*F* (1,35) = 6.33, *p* = .017, *η*_*p*_^2^ = 0.15). The interaction was influenced by a more significant disparity in response times between the valid and invalid cues when the task was difficult (*M*_*diff*_ = 53.94, 95%CI [33.67, 74.22]) compared to when it was easy (*M*_*diff*_ = 37.86, 95%CI [17.55, 58.09]).

#### Accuracy

**Fig. 11:**
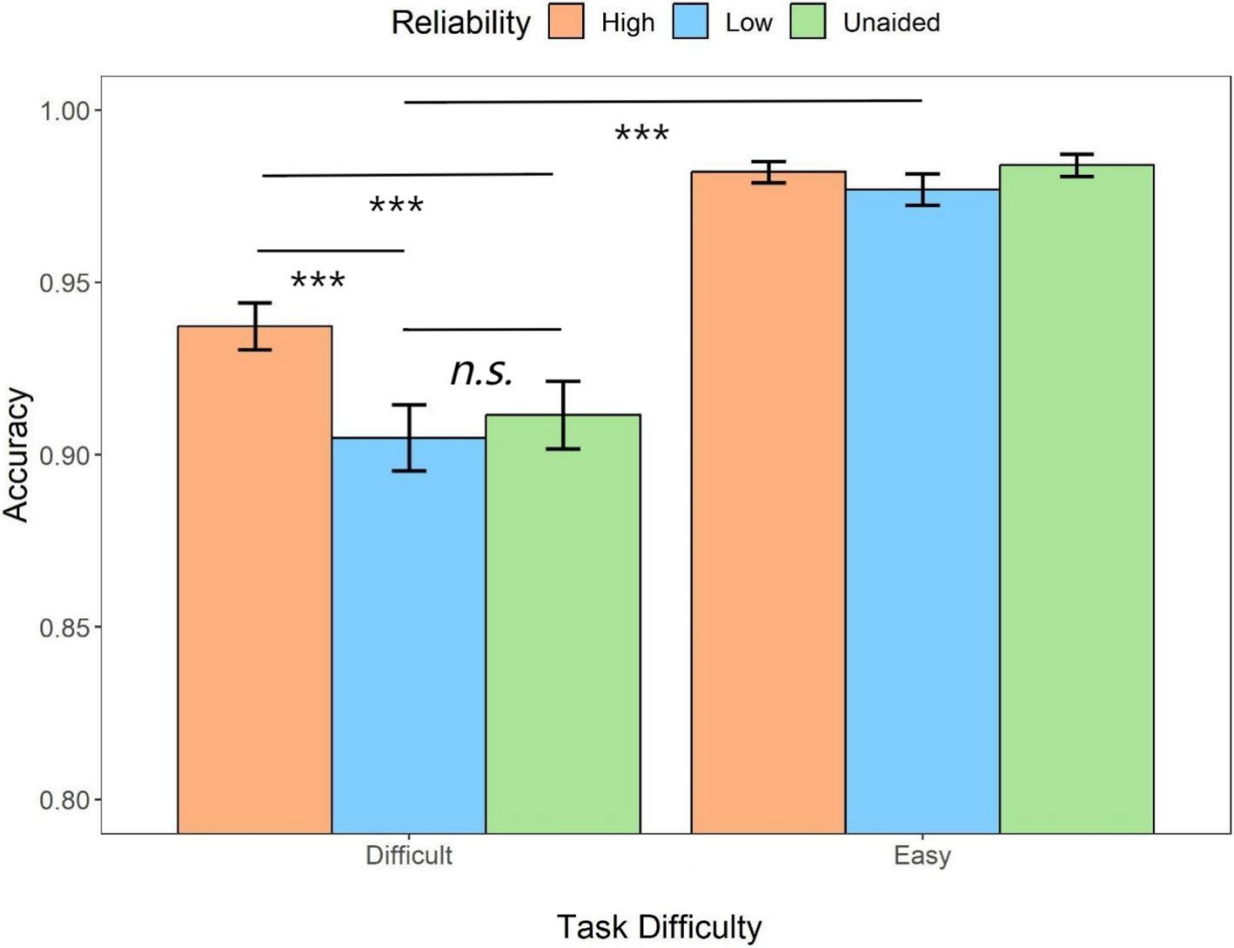
Mean accuracy across conditions of task difficulty (difficult, easy) and automation reliability (high, low, unaided) in Experiment 2. Error bars represent ± one standard-error

**Fig. 12:**
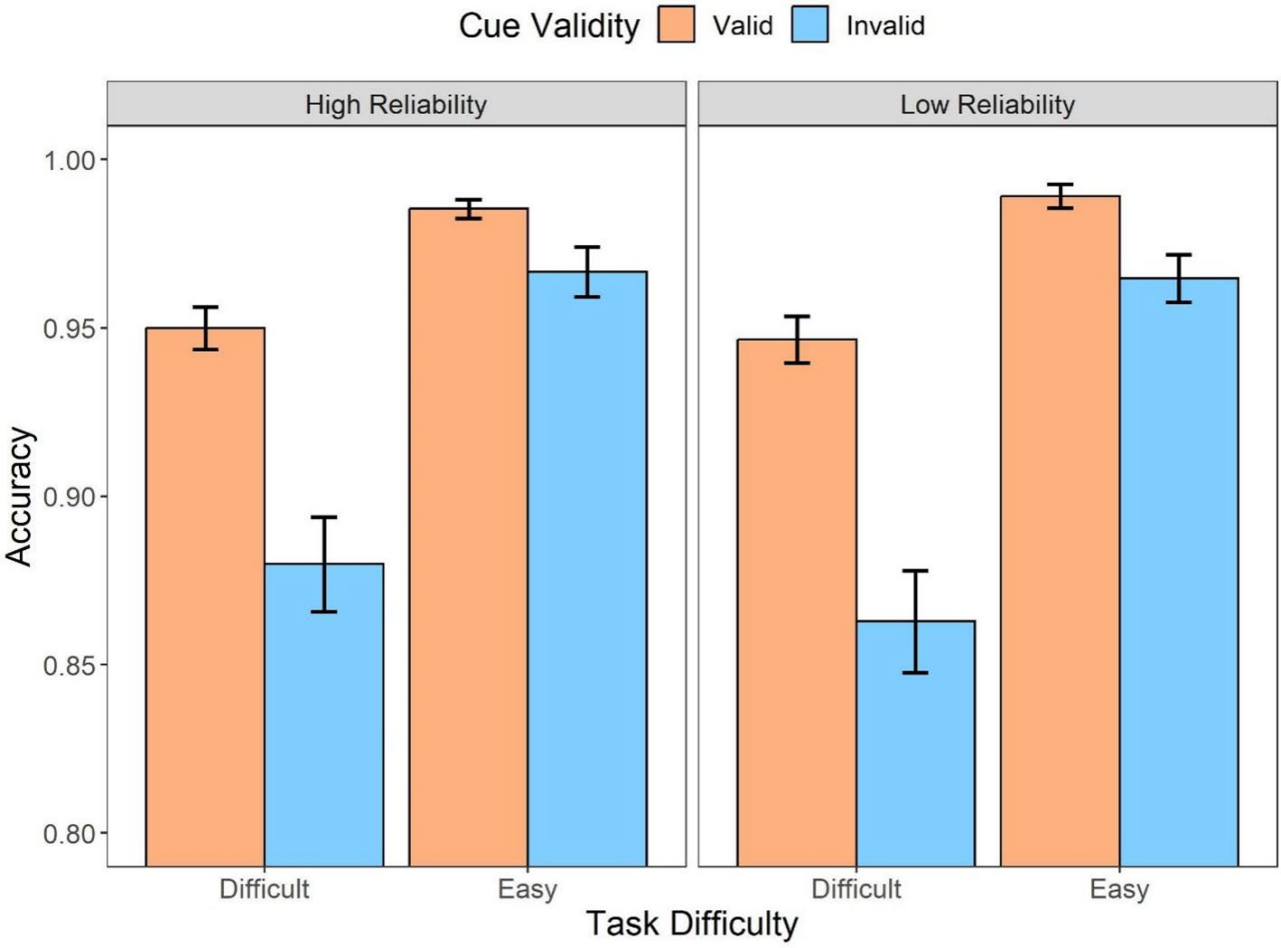
Mean accuracy across conditions of cue validity (valid, invalid) and task difficulty (difficult, easy) for different automation reliability (high, low) types in Experiment 2. Error bars represent ± one standard-error

As for response accuracy (Fig. 11), the repeated measures ANOVA analysis suggested that there was a main effect of the automation reliability (*F* (2, 70) = 17.00, *p* < .001, *η*_*p*_^2^ = 0.33). The high-reliability automation resulted in more accurate responses compared to the low-reliability automation (*t* = 5.76, *p* < .001, *d* = 0.46) and unaided responses (*t* = 3.67, *p* = .001, *d* = 0.29). There was no accuracy difference between the low-reliability and unaided responses (*t* = 2.10, *p* = .119, *d* = 0.17). In addition, there was also a main effect of the task difficulty (*F* (1,35) = 99.09, *p* < .001, *η*_*p*_^2^ = 0.74); the easy task resulted in more accurate responses compared to the difficult task (*t* = 9.95, *p* < .001, *d* = 1.55). The interaction between the two factors was also significant (*F* (2,70) = 18.06, *p* < .001, *η*_*p*_^2^ = 0.34): When the task was difficult, the high-reliability automation resulted in more accurate responses compared to low-reliability automation (*t* = 7.73, *p* < *.*001, *d* = 0.80) and unaided responses (*t* = 6.15, *p* < .001, *d* = 0.63) while there was no statistical significance between the low-reliability aids and unaided trials (*t* = 1.58, *p* = 1, *d* = 0.16); there was no statistical difference in accuracy among the three automated conditions when the task was easy (Fig. 12).

The automation information was further categorized into valid and invalid cues to evaluate the influence of cue validity on response accuracy. The analysis (Fig. 11) revealed that there was a main effect of the task difficulty (*F* (1,35) = 86.33, *p* < .001, *η*_*p*_^2^ = 0.71), in which the difficult task resulted in lower accuracy compared to the easy task (*t* = 9.29, *p* < .001, *d* = 1.24). The main effect of cue validity was also statistically significant (*F* (1,35) = 44.70, *p* < .001, *η*_*p*_^2^ = 0.56), suggesting that the trials with valid cues resulted in better performance compared to the trials with invalid cues (*t* = 6.69, *p* < .001, *d* = 0.92). In addition, there was also an interaction between cue validity and task difficulty (*F* (1,35) = 30.86, *p* < .001, *η*_*p*_^2^ = 0.47). The interaction was driven by the statistical significance between the valid and invalid cues when the task was difficult (*t* = 8.65, *p* < .001, *d* = 1.43) whereas such difference was not significant when the task was easy (*t* = 2.43, *p* = .11, *d* = 0.40).

#### Capacity coefficient

**Fig. 13:**
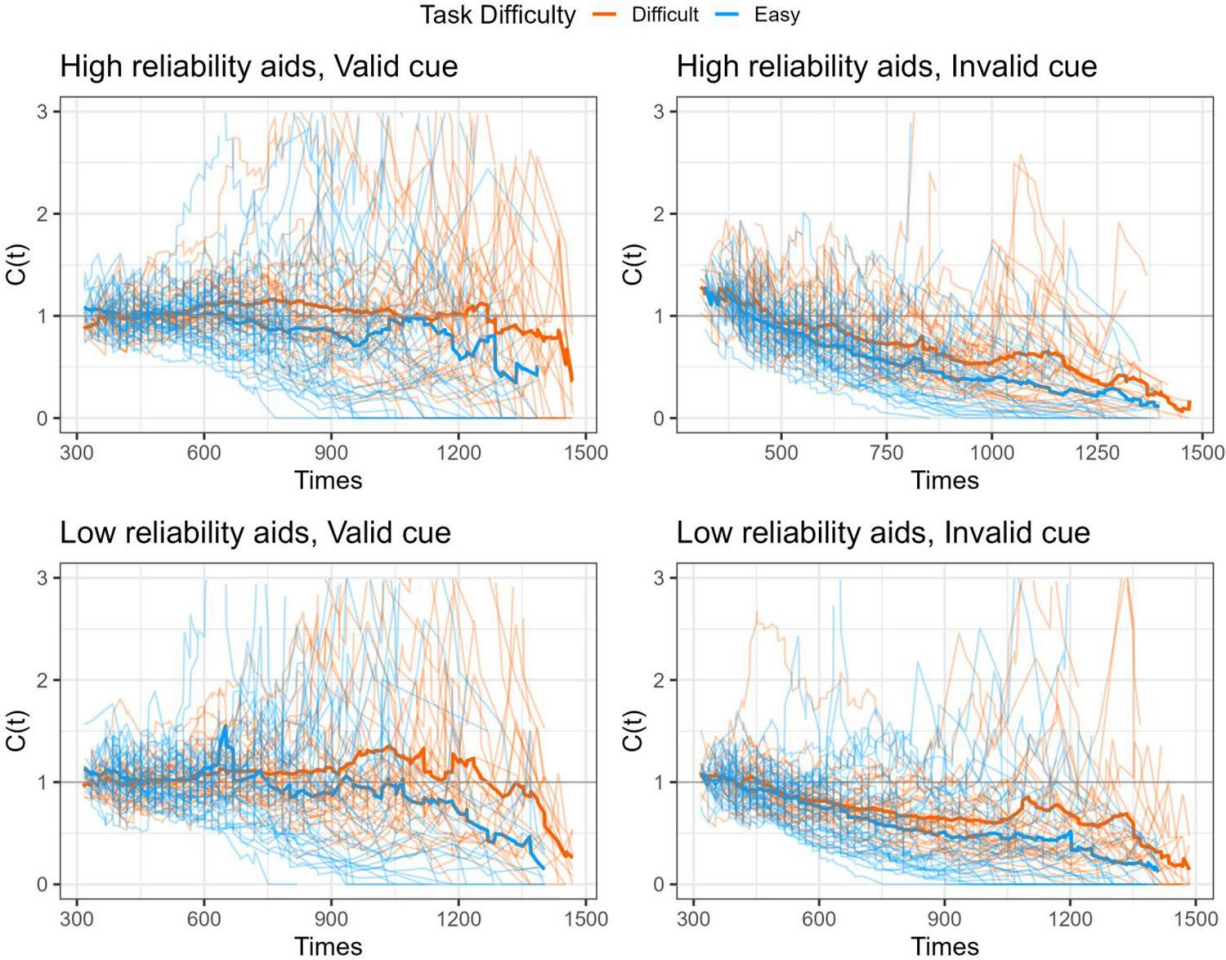
The temporal changes of capacity functions in Experiment 2. The thick line represents the average mean group level and the thin lines represent each individual’s capacity function

**Fig. 14:**
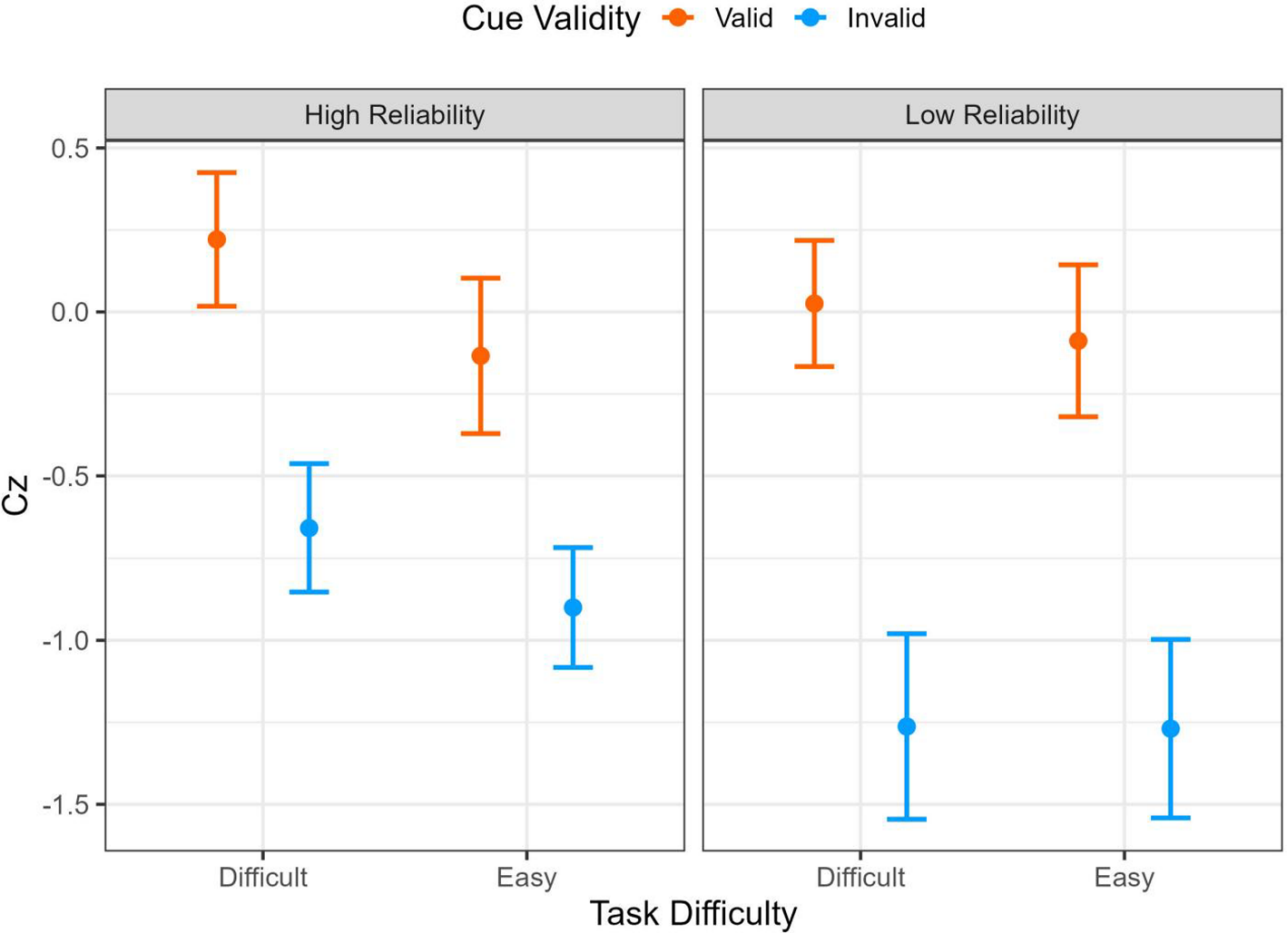
Mean Cz values for STST capacity coefficients for processing additional automated aids in Experiment 2. Cz at 0 implies unlimited capacity and below 0 implies limited capacity. The error bars represent ± one standard-error

The visual inspection of the STST capacity for Experiment 2 (Fig. 13) suggested that group performance was unlimited or limited capacity (below the *C*(t) = 1 baseline). In addition, there was no visually distinct difference between the two levels of task difficulty. The one-sample *t*-test suggests that when the cue was valid, the group performance was unlimited capacity regardless of the automation reliability and task difficulty; the Cz scores were not significantly different from 0. Nevertheless, participants performed at a limited capacity (i.e., Cz < 0) when the cue was invalid. The repeated measures ANOVA suggested that there was a main effect of the cue validity (*F* (1,35) = 21.75, *p* < .001, *η*_*p*_^2^ = 0.38), suggesting that the valid cue resulted in statistically more efficient processing compared to the invalid cues (*t* = 4.66, *p* < *.*001, *d* = 0.75). There was also a main effect of the automation reliability (*F* (1,35) = 5.37, *p* = .026, *η*_*p*_^2^ = 0.13); the high-reliability aid resulted in more efficient performance (*t* = 2.31, *p* = .026, *d* = 0.21). The results failed to find evidence suggesting the main effect of the task difficulty (*F* (1,35) = 0.77, *p* = .39, *η*_*p*_^2^ = 0.09 nor was there statistical significance for interactions among main effects (Fig. 14).

### Discussion

In Experiment 2, participants were instructed to perform a shape categorization task under intermixed automation conditions. Unlike Experiment 1, in which high and low automation reliability resulted in different behavioral performances, Experiment 2 indicated that the random presentation of different types of automation reliability in an intermixed design did not yield a significant reliability effect on both response times and accuracy. The findings indicated that the impact of reliability was likely replaced by cue validity, as the introduction of valid cues led to increased accuracy and faster responses. Furthermore, an interaction was observed between cue validity and task difficulty. In difficult tasks, notable differences emerged between responses influenced by valid and invalid cues, both in terms of response times and accuracy. This indicates that invalid cues presented a greater challenge for participants in their responses.

Unlike Experiment 1, the STST analysis indicated that participants’ performance capacity was limited when the automated aids delivered invalid cues, regardless of the reliability type. This disparity is likely due to participants’ difficulty in distinguishing between high- and low-reliability automated aids when limited by cognitive resources. This may also indicate that participants tend to disregard information when its reliability is uncertain. Consequently, instead of selectively assessing the dependability of automation, participants may focus on evaluating the validity of information in each trial.

Furthermore, Experiment 2 did not show the beneficial use of automated aids in difficult tasks, suggesting that the repeated display of automation, as in Experiment 1, was essential for participants to allocate attention resources to automation that enhanced the task performance in difficult tasks. In addition, our evidence indicated an interaction between cue validity and automation reliability, where the difference in decision efficiency between valid and invalid cues was more pronounced in trials supported by high-reliability automation. This difference may be attributed to an uneven number of cues associated with various types of reliability automation, as high-reliability automation offers a greater number of valid cues. Considering that cue validity may influence participants’ attention (e.g., Horowitz, 2017), we propose that future research should investigate this specific response tendency further.

## General discussion

The impact of task difficulty and automation reliability played a crucial role in shaping participants’ use of automated assistance. Participants were more likely to seek assistance from automation during difficult tasks and were more influenced by it; nevertheless, when the automation was unreliable, it could become a hindrance and potentially have a negative impact on performance (e.g., Wohleber et al., 2016). In our current study, participants were instructed to categorize the shape of the stimulus. Experiment 1 had participants perform the task under one specific type of automation condition (low reliability, high reliability, unaided) at a time, whereas Experiment 2 instructed participants to perform the task with an intermixed design in which the three types of automation were presented randomly. We analyzed the data using STST capacity analysis, comparing participants’ performance against a baseline established by participants’ performance in the unaided condition. This allows us to measure the decision efficiency of human information processing with automated aids. The findings indicated that when the automation provided valid cues, participants processed information more efficiently than when given invalid cues. Furthermore, the efficiency was enhanced when automation aided in difficult tasks, as demonstrated in Experiment 1. By contrast, this efficiency gain was not as apparent in Experiment 2, where participants encountered varying types of automated aids randomly. Furthermore, the temporal analysis of the capacity coefficient in Experiment 1 revealed that individuals had super capacity during the 500ms and 750 ms intervals, underscoring the dynamic fluctuations in decision efficiency throughout the response period.

Our STST capacity analysis indicated that automated aids are beneficial in difficult tasks, especially when participants could distinctly discern between low- and high-reliability automation. Nevertheless, this discovery contrasts with the findings of Yamani and McCarley (2018), who found that the easier task resulted in more efficient processing. This distinction depends on whether the responses of automated aids are seen as independent or an essential component of the decision-making process (Parasuraman et al., 2000). Our investigation revealed that when information was abundant for decision-making and participants could consider aided suggestions, they exhibited unlimited capacity when the aids provided correct recommendations. This aligns with the findings of Kneeland et al. (2021), which demonstrated that participants displayed similar, if not inferior, efficiency with the automated aids compared to independent decision-making, despite no observed impact of difficulty.

Kneeland et al. (2024) observed that participants generally processed automated assistance and raw stimulus information sequentially, often neglecting to incorporate both into their decision-making considerations. Participants did not consistently rely solely on automated aids; rather, they seemed to switch between both sources across trials. This may partially explain our observation in Experiment 2, where the reliability of automation in an intermixed block design led participants to allocate attentional resources toward verifying the cues’ validity rather than accumulating reliability information for each presented automated aid. An alternative explanation is that when automation is presented in an intermixed block design, participants may struggle to form consistent expectations regarding the automation’s reliability. Following the expectancy violation theory (Burgoon, 2016), Fox et al. (2025) found that differences in team efficiency between low- and high-purported reliability teammates were moderated by whether the teammates’ actual performance aligned with expectations. Consistent with research on collaborative decision-making, this suggests the potential for induced coordination costs, as participants must synchronize their efforts (e.g., Fific & Gigerenzer, 2014; Neider et al., 2010; Yamani et al., 2017; Zhang et al., 2025). In the context of aided decision-making, decision makers were also less likely to respond to or assign weight to information from the automation when it was perceived to have low informative value (Maltz & Meyer, 2001; Meyer, 2001).

Together, our results suggested that complacency regarding highly reliable automation may be partially ascribed to the validity of information, represented by a weighted trade-off between decision makers’ belief in their performance and automation capability. Accordingly, we propose that future research in aided decision-making should ensure that decision makers are fully informed of the automation’s limitations to mitigate the complacency effect. Furthermore, considering the difficulties encountered by participants in dynamic decision-making contexts (e.g., Cronin et al., 2009; Gonzalez, 2004), it is essential for future research to elucidate the manner in which information regarding automation capability is accumulated on a trial-by-trial basis (e.g., Gonzalez et al., 2003; Gonzalez & Dutt, 2011), in conjunction with various influential factors (Lyons & Stokes, 2012).

In contrast to previous research where low-reliability automated aids resulted in performance costs (Wickens & Dixon, 2007), in our current study, participants had processing efficiency that was equally likely with low-reliability automated aids, as it provided accurate cues. We suspected that this could be accounted for by our limitation in the current research, which only adopted a restricted set of stimuli, and the range of task difficulty levels is limited. Additionally, the likelihood of valid cues was directly tied to the automation’s reliability. To address such limitations, future research can benefit from employing a broader array of stimulus variations (e.g., Kneeland et al., 2021), various automated decision-making stages (Rovira et al., 2002), and automated aids that indicate the probability of a stimulus belonging to a certain category (e.g., Bergert & Nosofsky, 2007). Furthermore, Dixon et al. (2007) proposed that automation prone to false alarms and misses might influence participants’ interactions with automated aids differently. Specifically, they suggested that false alarm-prone automation impacts both decision makers’ compliance (action following automation’s signal diagnosis) and reliance (action following automation’s noise diagnosis) whereas miss-prone automation seemed to affect only reliance. In our current research, we oversimplified by using overall accuracy as the measure of automation reliability and our design of the automated aids display differs from previous research in human-automation interaction (e.g., Kneeland et al., 2021). All can contribute to variations in participants’ cognitive abilities in our current research. In this way, future research can explore whether participants adopt different strategies when faced with automated aids that exhibit various types of errors in a different display design, providing deeper insights into how decision makers interact with and adapt to automation in decision-making process.

To conclude, our research explored the impact of task difficulty on processing efficiency by automation reliability. Our results using the STST capacity coefficient in SFT suggested that participants performed the categorization task comparably with or without the presence of automated aids, provided they were not distracted by invalid cues. In accordance with our hypothesis, participants exhibited a significant advantage when engaged in difficult categorization tasks with the assistance of automated aids. The advantageous condition observed in the difficult task became apparent when participants interacted with a singular sort of automation, thereby accumulating reliable information for that automation. Our research suggested the existence of an optimal scenario for employing automation to aid decision makers and can contribute to interface design to optimize processing efficiency inside the established automation system.

## Appendix

The overall reliability of the automation was 82% in the high-reliability condition and 50% in the low-reliability condition. Each block consisted of 4 sets of 100 trials, with each stimulus (see Fig. 1) presented 25 times, totaling 400 trials per block (i.e., 200 trials for category A and 200 for category B).

For every 100 trials involving category A:

- In the high reliability condition, the automation provided 82 valid suggestions and 18 invalid ones. Of these, 1 out of the 82 valid suggestions and 9 out of the 18 invalid suggestions were accompanied by incorrect feedback (see Footnote 1 for details).
- In the low-reliability condition, the automation provided 50 valid and 50 invalid suggestions, with 5 of each suggestion receiving incorrect feedback.

See Table 1.

**Table 1:** Automation design example for each set of 100 trials with category A

Advised				
Feedback	High reliability		Low reliability	
	A	B	A	B
A	**81**	**1**	**45**	**5**
B	9	9	45	5

The correct answers (i.e., valid information) were in bold

## Footnotes

1 To test the aid reliance in the context of decision under uncertainty, participants received 90% “correct” feedback and 10% “incorrect” feedback when they made correct responses, the same applies to when participants made incorrect responses in the current design. We expected that this design would help participants adjust their reliance on automation and encourage participants to process the stimuli information. See the Appendix for more detailed explanations.

## References

[CR1] Barg-Walkow, L. H., & Rogers, W. A. (2016). The effect of incorrect reliability information on expectations, perceptions, and use of automation. *Human Factors,**58*(2), 242–260. 10.1177/001872081561027 26519483 10.1177/0018720815610271 PMC10664720

[CR2] Bergert, F. B., & Nosofsky, R. M. (2007). A response-time approach to comparing generalized rational and take-the-best models of decision making. *Journal of Experimental Psychology: Learning, Memory, and Cognition,**33*(1), 107. 17201556 10.1037/0278-7393.33.1.107

[CR500] Blaha, L. M., Townsend, J. T., Kneeland, C. M., & Houpt, J. W. (Under Review). Capacity coefficient analysis for single-target selfterminating processes. *Journal of Mathematical Psychology*.

[CR3] Bowers, C., Thornton, C., Braun, C., Morgan, B. B., Jr., & Salas, E. (1998). Automation, task difficulty, and aircrew performance. *Military Psychology,**10*(4), 259–274. 11541776 10.1207/s15327876mp1004_3

[CR4] Burgoon, J. K. (2016). Expectancy violations theory. In C. R. Berger, M. E. Roloff, S. R. Wilson, J. P. Dillard, J. Caughlin, & D. Solomon (Eds.), *International encyclopedia of interpersonal communication* (pp. 1–9). Wiley.

[CR5] Chen, J., Mishler, S., Hu, B., Li, N., & Proctor, R. W. (2018). The description-experience gap in the effect of warning reliability on user trust and performance in a phishing-detection context. *International Journal of Human-Computer Studies,**119*, 35–47.

[CR6] Cronin, M. A., Gonzalez, C., & Sterman, J. D. (2009). Why don’t well-educated adults understand accumulation? A challenge to researchers, educators, and citizens. *Organizational Behavior and Human Decision Processes,**108*(1), 116–130.

[CR7] De Vries, P., Midden, C., & Bouwhuis, D. (2003). The effects of errors on system trust, self-confidence, and the allocation of control in route planning. *International Journal of Human-Computer Studies,**58*(6), 719–735.

[CR8] Dixon, S. R., Wickens, C. D., & McCarley, J. S. (2007). On the independence of compliance and reliance: Are automation false alarms worse than misses? *Human Factors,**49*(4), 564–572. 17702209 10.1518/001872007X215656

[CR9] Dunning, D., Johnson, K., Ehrlinger, J., & Kruger, J. (2003). Why people fail to recognize their own incompetence. *Current Directions in Psychological Science,**12*(3), 83–87. 10.1111/1467-8721.01235

[CR10] Dzindolet, M. T., Peterson, S. A., Pomranky, R. A., Pierce, L. G., & Beck, H. P. (2003). The role of trust in automation reliance. *International Journal of Human-Computer Studies,**58*(6), 697–718. 10.1016/S1071-5819(03)00038-7

[CR11] Fifić, M., & Gigerenzer, G. (2014). Are two interviewers better than one? *Journal of Business Research,**67*(8), 1771–1779. 10.1016/j.jbusres.2014.03.003

[CR12] Fox, E. L., Capiola, A., Bowers, G., & Stephenson, A. (2025). A metric of team multitasking throughput. *Journal of Experimental Psychology: Applied,**31*(1), 58–70. 10.1037/xap0000519 39680014 10.1037/xap0000519

[CR13] Gamble, K. R., Cassenti, D. N., & Buchler, N. (2018). Effects of information accuracy and volume on decision making. *Military Psychology,**30*(4), 311–320.

[CR14] Gonzalez, C. (2004). Learning to make decisions in dynamic environments: Effects of time constraints and cognitive abilities. *Human Factors,**46*(3), 449–460. 15573545 10.1518/hfes.46.3.449.50395

[CR15] Gonzalez, C., & Dutt, V. (2011). Instance-based learning: Integrating sampling and repeated decisions from experience. *Psychological Review,**118*(4), 523–551. 10.1037/a0024558 21806307 10.1037/a0024558

[CR16] Gonzalez, C., Lerch, J. F., & Lebiere, C. (2003). Instance-based learning in dynamic decision making. *Cognitive Science,**27*(4), 591–635.

[CR17] Hoff, K. A., & Bashir, M. (2015). Trust in automation: Integrating empirical evidence on factors that influence trust. *Human Factors,**57*(3), 407–434. 10.1177/0018720814547570 25875432 10.1177/0018720814547570

[CR18] Horowitz, T. S. (2017). Prevalence in visual search: From the clinic to the lab and back again. *Japanese Psychological Research,**59*(2), 65–108.

[CR501] Houpt, J. W., & Townsend, J. T. (2012). Statistical measures for workload capacity analysis. *Journal of Mathematical Psychology, 56*(5), 341–355. 10.1016/j.jmp.2012.05.004 PMC3501136 23175582

[CR19] Houpt, J. W., Blaha, L. M., McIntire, J. P., Havig, P. R., & Townsend, J. T. (2013). Systems factorial technology with R. *Behavior Research Methods,**46*(2), 307–330. 10.3758/s13428-013-0377-3 10.3758/s13428-013-0377-3 24019062

[CR20] JASP Team. (2024). JASP (Version 0.19.3) [Computer software].

[CR21] Kantowitz, B. H., Hanowski, R. J., & Kantowitz, S. C. (1997). Driver acceptance of unreliable traffic information in familiar and unfamiliar settings. *Human Factors,**39*(2), 164–176. 10.1518/001872097778543831

[CR22] Kneeland, C. M., Houpt, J. W., & Bennett, K. B. (2021). Exploring the performance consequences of target prevalence and ecological display designs when using an automated aid. *Computational Brain & Behavior,**4*, 335–354.

[CR23] Kneeland, C. M., Houpt, J. W., & Juvina, I. (2024). How do people process information from automated decision aids: An application of systems factorial technology. *Computational Brain & Behavior,**7*(1), 106–128. 10.1007/s42113-023-00188-z 10.1007/s42113-023-00188-z PMC13292819 42459521

[CR24] Lee, J. D., & See, K. A. (2004). Trust in automation: Designing for appropriate reliance. *Human Factors,**46*(1), 50–80. 10.1518/hfes.46.1.50_30392 15151155 10.1518/hfes.46.1.50_30392

[CR25] Lee, J., & Moray, N. (1992). Trust, control strategies and allocation of function in human-machine systems. *Ergonomics,**35*(10), 1243–1270. 1516577 10.1080/00140139208967392

[CR26] Little, D., Altieri, N., Fific, M., & Yang, C. T. (Eds.). (2017). *Systems factorial technology: A theory driven methodology for the identification of perceptual and cognitive mechanisms*. Academic Press.

[CR27] Lyons, J. B., & Stokes, C. K. (2012). Human-human reliance in the context of automation. *Human Factors,**54*(1), 112–121. 22409106 10.1177/0018720811427034

[CR28] Madhavan, P., Wiegmann, D. A., & Lacson, F. C. (2006). Automation failures on tasks easily performed by operators undermine trust in automated aids. *Human Factors,**48*(2), 241–256. 10.1518/001872006777724408 16884046 10.1518/001872006777724408

[CR29] Maltz, M., & Meyer, J. (2001). Use of warnings in an attentionally demanding detection task. *Human Factors: THe Journal of the Human Factors and Ergonomics Society,**43*(2), 217–226. 10.1518/001872001775900931 10.1518/001872001775900931 11592663

[CR30] Merritt, S. M., Lee, D., Unnerstall, J. L., & Huber, K. (2015). Are well-calibrated users effective users? Associations between calibration of trust and performance on an automation-aided task. *Human Factors,**57*(1), 34–47. 10.1177/0018720814561675 25790569 10.1177/0018720814561675

[CR31] Metzger, U., & Parasuraman, R. (2005). Automation in future air traffic management: Effects of decision aid reliability on controller performance and mental workload. *Human Factors,**47*(1), 35–49. 10.1518/0018720053653802 15960085 10.1518/0018720053653802

[CR32] Meyer, J. (2001). Effects of warning validity and proximity on responses to warnings. *Human Factors,**43*(4), 563–572. 10.1518/001872001775870395 12002005 10.1518/001872001775870395

[CR33] Neider, M. B., Chen, X., Dickinson, C. A., Brennan, S. E., & Zelinsky, G. J. (2010). Coordinating spatial referencing using shared gaze. *Psychonomic Bulletin & Review,**17*, 718–724. 10.3758/PBR.17.5.718 21037172 10.3758/PBR.17.5.718

[CR34] Parasuraman, R., & Wickens, C. D. (2017). Humans: Still vital after all these years of automation. *Decision Making in Aviation* (pp. 251–260). Routledge. 10.1518/001872008X312198 18689061

[CR35] Parasuraman, R., Sheridan, T. B., & Wickens, C. D. (2000). A model for types and levels of human interaction with automation. *IEEE Transactions on Systems, Man, and Cybernetics-Part a: Systems and Humans,**30*(3), 286–297. 10.1109/3468.844354 11760769

[CR36] Patton, C. E. (2023). *The influence of trust, self-confidence and task difficulty on automation use*. Doctoral dissertation, Colorado State University.

[CR37] Pop, V. L., Shrewsbury, A., & Durso, F. T. (2015). Individual differences in the calibration of trust in automation. *Human Factors,**57*(4), 545–556. 10.1177/0018720814564422 25977317 10.1177/0018720814564422

[CR38] Rieger, T., & Manzey, D. (2022). Human performance consequences of automated decision aids: The impact of time pressure. *Human Factors,**64*(4), 617–634. 10.1177/0018720820965019 33111557 10.1177/0018720820965019

[CR39] Rovira, E., McGarry, K., & Parasuraman, R. (2002). Effects of unreliable automation on decision making in command and control. In *Proceedings of the Human Factors and Ergonomics Society Annual Meeting* (Vol. 46, No. 3, pp. 428–432). SAGE Publications.

[CR40] Rovira, E., McGarry, K., & Parasuraman, R. (2007). Effects of imperfect automation on decision making in a simulated command and control task. *Human Factors,**49*(1), 76–87. 17315845 10.1518/001872007779598082

[CR41] Schaefer, K. E., Chen, J. Y. C., Szalma, J. L., & Hancock, P. A. (2016). A meta-analysis of factors influencing the development of trust in automation: Implications for understanding autonomy in future systems. *Human Factors,**58*(3), 377–400. 10.1177/0018720816634228 27005902 10.1177/0018720816634228

[CR42] Schwark, J., Dolgov, I., Graves, W., & Hor, D. (2010). The influence of perceived task difficulty and importance on automation use. In *Proceedings of the Human Factors and Ergonomics Society Annual Meeting* (Vol. 54, No. 19, pp. 1503–1507). SAGE Publications.

[CR43] Sheridan, T. B., & Parasuraman, R. (2005). Human-automation interaction. *Reviews of Human Factors and Ergonomics,**1*(1), 89–129.

[CR44] Townsend, J. T., & Nozawa, G. (1995). Spatio-temporal properties of elementary perception: An investigation of parallel, serial, and coactive theories. *Journal of Mathematical Psychology,**39*(4), 321–359. 10.1006/jmps.1995.1033

[CR45] Wickens, C. D. (2002). Multiple resources and performance prediction. *Theoretical Issues in Ergonomics Science,**3*(2), 159–177. 10.1080/14639220210123806

[CR46] Wickens, C. D., & Dixon, S. R. (2007). The benefits of imperfect diagnostic automation: A synthesis of the literature. *Theoretical Issues in Ergonomics Science,**8*(3), 201–212. 10.1080/14639220110110306

[CR47] Wickens, C. D. (2000). Imperfect and unreliable automation and its implications for attention allocation, information access and situation awareness. Technical Report ARL00-l OMASA-00-2.

[CR48] Wiegmann, D. A., Rich, A., & Zhang, H. (2001). Automated diagnostic aids: The effects of aid reliability on users’ trust and reliance. *Theoretical Issues in Ergonomics Science,**2*(4), 352–367.

[CR49] Wohleber, R. W., Calhoun, G. L., Funke, G. J., Ruff, H., Chiu, C. Y. P., Lin, J., & Matthews, G. (2016). The impact of automation reliability and operator fatigue on performance and reliance. In *Proceedings of the Human Factors and Ergonomics Society Annual Meeting* (Vol. 60, No. 1, pp. 211–215). SAGE Publications.

[CR50] Xu, X., Wickens, C. D., & Rantanen, E. M. (2007). Effects of conflict alerting system reliability and task difficulty on pilots’ conflict detection with cockpit display of traffic information. *Ergonomics,**50*(1), 112–130. 17178655 10.1080/00140130601002658

[CR51] Yamani, Y., & McCarley, J. S. (2018). Effects of task difficulty and display format on automation usage strategy: A workload capacity analysis. *Human Factors,**60*(4), 527–537. 29470135 10.1177/0018720818759356

[CR52] Yamani, Y., Neider, M. B., Kramer, A. F., & McCarley, J. S. (2017). Characterizing the efficiency of collaborative visual search with systems factorial technology. *Archives of Scientific Psychology,**5*(1), 1–9. 10.1037/arc0000030

[CR53] Zhang, H., Zhu, P. F., & Yang, C. T. (2025). Group efficiency based on the termination rule in the multiple-targets visual search task. *Psychonomic Bulletin & Review,**32*(1), 463–471. 39147959 10.3758/s13423-024-02558-5

